# Genetic risk factors for colorectal cancer in multiethnic Indonesians

**DOI:** 10.1038/s41598-021-88805-4

**Published:** 2021-05-11

**Authors:** Irawan Yusuf, Bens Pardamean, James W. Baurley, Arif Budiarto, Upik A. Miskad, Ronald E. Lusikooy, Arham Arsyad, Akram Irwan, George Mathew, Ivet Suriapranata, Rinaldy Kusuma, Muhamad F. Kacamarga, Tjeng W. Cenggoro, Christopher McMahan, Chase Joyner, Carissa I. Pardamean

**Affiliations:** 1 Faculty Medicine, Hasanuddin University, Makassar, South Sulawesi Indonesia; 2 Bioinformatics & Data Science Research Center, Bina Nusantara University, Jakarta, DKI Jakarta Indonesia; 3 Mochtar Riady Institute for Nanotechnology, Pelita Harapan University, Tangerang, Banten Indonesia; 4 Computer Science Department, BINUS Graduate Program-Master of Computer Science Program, Bina Nusantara University, Jakarta, DKI Jakarta Indonesia; 5 Computer Science Department, School of Computer Science, Bina Nusantara University, Jakarta, DKI Jakarta Indonesia; 6 School of Mathematical and Statistical Sciences, Clemson University, Clemson, SC USA

**Keywords:** Genome-wide association studies, Cancer genomics

## Abstract

Colorectal cancer is a common cancer in Indonesia, yet it has been understudied in this resource-constrained setting. We conducted a genome-wide association study focused on evaluation and preliminary discovery of colorectal cancer risk factors in Indonesians. We administered detailed questionnaires and collecting blood samples from 162 colorectal cancer cases throughout Makassar, Indonesia. We also established a control set of 193 healthy individuals frequency matched by age, sex, and ethnicity. A genome-wide association analysis was performed on 84 cases and 89 controls passing quality control. We evaluated known colorectal cancer genetic variants using logistic regression and established a genome-wide polygenic risk model using a Bayesian variable selection technique. We replicate associations for rs9497673, rs6936461 and rs7758229 on chromosome 6; rs11255841 on chromosome 10; and rs4779584, rs11632715, and rs73376930 on chromosome 15. Polygenic modeling identified 10 SNP associated with colorectal cancer risk. This work helps characterize the relationship between variants in the *SCL22A3*, *SCG5*, *GREM1*, and *STXBP5-AS1* genes and colorectal cancer in a diverse Indonesian population. With further biobanking and international research collaborations, variants specific to colorectal cancer risk in Indonesians will be identified.

## Introduction

Colorectal cancer is one of the most common cancers in the world and a leading cause of cancer-related deaths^1,2^. There is growing evidence that colorectal cancer rates are changing in Asian countries, but the causes are still under investigation^3,4^. Colorectal cancer is now one of the top three cancers in many Asian countries^4^. Currently, Asia contributes to 48% of the total number of new colorectal cancer cases in the world, of which the majority are found in Eastern Asia^5^. Specifically in Indonesia, the age-standardized incidence for males and females has been reported as 15.9 and 10.1 per 100,000 respectively^6^.

The heritability of colorectal cancer is estimated to be between 12 and 35%. However, germline mutations that are highly penetrant contribute less than 5% to colorectal cancer^7^. Nonetheless, increasing evidence is finding that heritability plays a potential, crucial role in colorectal cancer pathogenesis. Currently, mutations in 14 genes are suspected to underlie different subtypes of colorectal cancer, including mutations in the APC that increases predisposition to familial adenomatous polyposis (FAP) and defects in mismatch repair genes associated with Lynch Syndrome^7^. Recent genome-wide association studies have identified common genetic variants linked to colorectal cancer predisposition, highlighting a greater association between heritable risk and the disease. Thus far, over 40 genetic variants have been identified, within several well-known biological pathways that have been shown to be highly relevant to oncogenesis, including the TGF-beta/BMP pathway and the mitogen-activated protein kinases (MAPK) pathway^7^.

However, many of these colorectal cancer genetic associations were discovered in European-ancestry populations but do not replicate well in other ancestry groups, demonstrating the need for studies in diverse populations worldwide^8^. The Asia Colorectal Cancer Consortium was initiated in 2009 among East Asian nations and has successfully identified novel relevant, genetic regions^9,10^. However, colorectal cancer cases from South East Asian cohorts have been under represented.

Given the changes in colorectal cancer rates in Asia and the differences in risk factors present in ethnically diverse South East Asia, we present results of the first genomic association study of colorectal cancer in Indonesia. We present results from the initial phase of this study, focused on cases from South Sulawesi, Indonesia.

## Results

### Characteristics of study sample

The characteristics of the colorectal cancer cases and controls are summarized in Table 1. The mean age of the colorectal cancer cases was 54 years. The majority of cases were male (57%). Among ethnicities, most cases were self-reported Bugis (44%) or Makassar ethnicity (27%). Controls appeared to be adequately frequency matched to cases by age, sex, and ethnicity ($p > 0.05$). Colorectal cancer cases had lower average body mass index (BMI) and were more likely to be smokers than controls ($p < 0.01$). Estimated genetically, the majority of both cases and controls were of East Asian ancestry. 82% of the cases had late stage cancer (III or IV) which unfortunately is consistent with recent reports in Indonesia^11^. As seen in other studies, the most common colorectal cancer site was rectum (43%)^12,13^.

**Table 1:** Characteristics of South Sulawesi colorectal cancer cases and controls.

	Cases	Controls	P
	N = 89	N = 84	
Age	53.8 (13.2)	50.5 (14.5)	0.12
**Gender**			> 0.99
Female	38 (42.7%)	36 (42.9%)	
Male	51 (57.3%)	48 (57.1%)	
**Ethnicity**			0.68
Bugis	39 (43.8%)	45 (53.6%)	
Makassar	24 (27.0%)	23 (27.4%)	
Mandar	2 (2.3%)	1 (1.2%)	
Toraja	10 (11.2%)	8 (9.5%)	
Non South Sulawesi	9 (10.1%)	4 (4.8%)	
Non Sulawesi	5 (5.6%)	3 (3.6%)	
BMI	21.2 (3.1)	24.5 (3.6)	< 0.01
**Smoking status**			< 0.01
Smoker	39 (43.8%)	15 (17.9%)	
Non smoker	50 (56.2%)	69 (82.1%)	
**Ancestry (estimated)**			
East Asian (EAS)	0.92	0.94	0.02
South Asian (SAS)	0.07	0.05	0.15
African (AFR)	< 0.01	< 0.01	0.02
European (EUR)	0.01	0.01	0.36
**Cancer site**			
Right colon	15 (16.9%)	–	
Transversum	9 (10.1%)	–	
Left colon	1 (1.12%)	–	
Sigmoid Rectum	26 (29.2%)	–	
	38 (42.7%)	–	
**Staging**			
I	3 (3.4%)	–	
II	9 (10.1%)	–	
III	62 (69.7%)	–	
IV	11 (12.4%)	–	

### Genome-wide association analysis

As expected given the sample size, no SNPs met the historical cutoff set for genome-wide significance (Supplementary Figs. 6 and 7). The summaries for all variants with a marginal p-value < 5E 5 are included in the “Supplementary materials” (Table 4). These include two intergenic SNPs and two SNPs in the *MRO* gene on chromosome 18.

**Figure 1:**
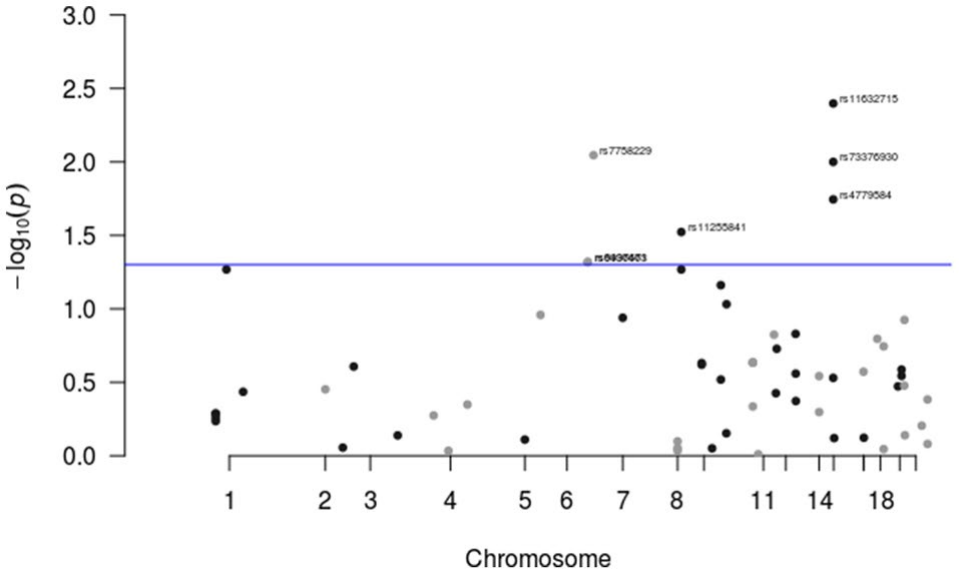
Results for known colorectal cancer susceptibility SNPs. Variants with p-values $< 0.05$ were flagged for further investigation.

**Figure 2:**
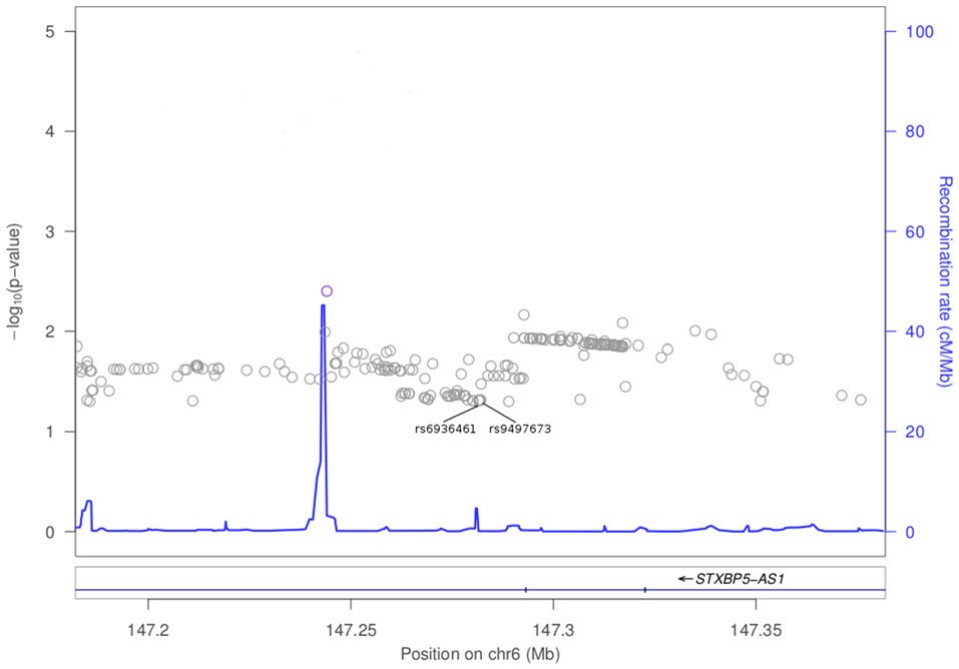
Association plot for 100 kb region flanking rs6936461 on chromosome 6.

**Figure 3:**
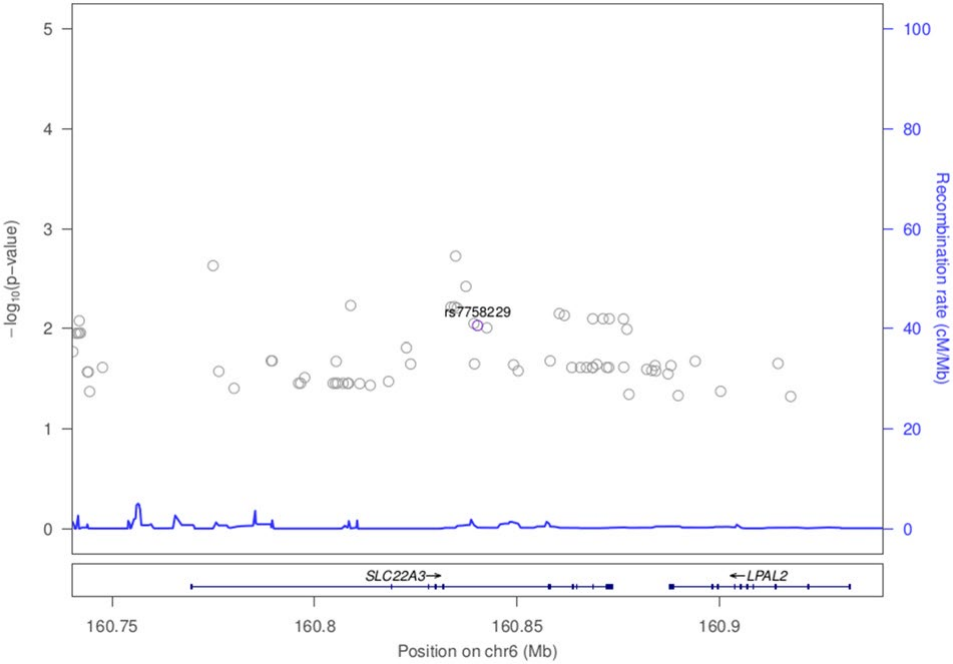
Association plot for 100 kb region flanking rs7758229 on chromosome 6.

**Figure 4:**
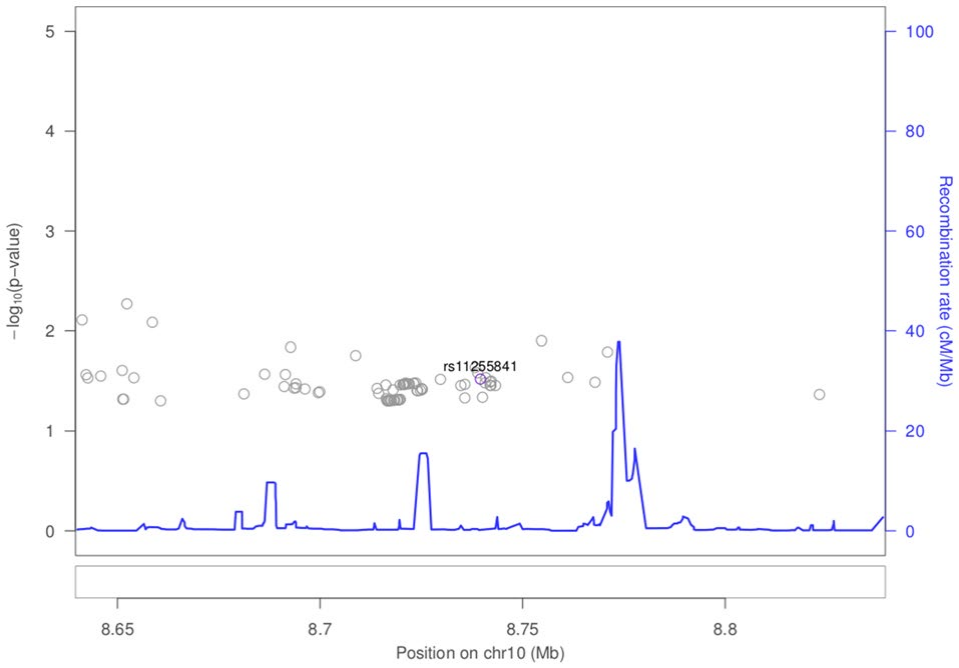
Association plot for 100 kb region flanking rs11255841 on chromsome 10.

**Figure 5:**
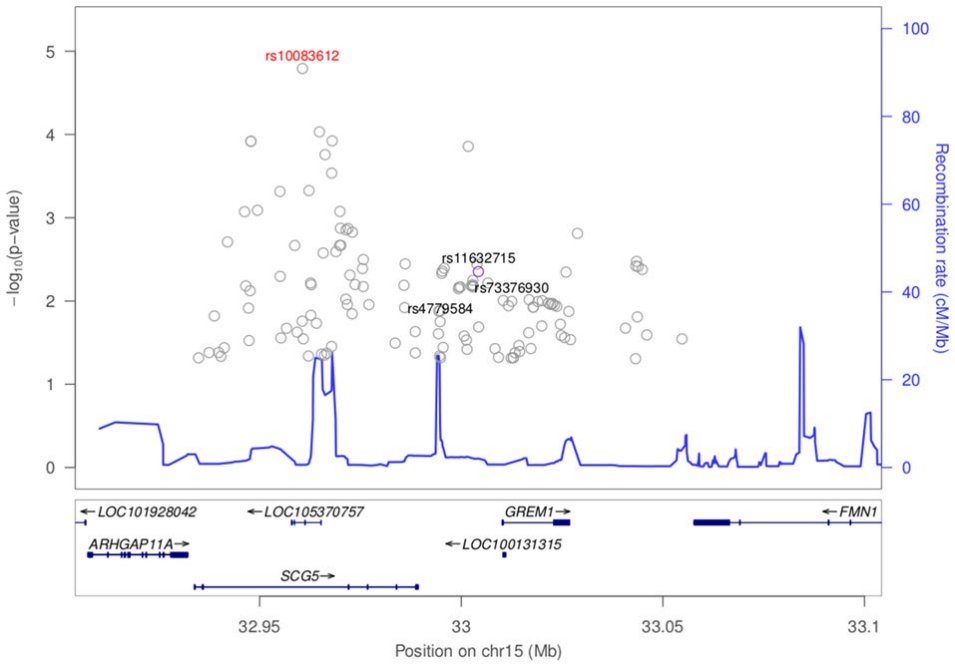
Association plot for 100 kb flanking rs11632715 on chromosome 15. The top associated SNP in the region was rs10083612.

Results for previously reported colorectal cancer SNPs are presented in Fig. 1 and Supplementary Table 3. There is evidence of replication for the following genetic variants: rs9497673, rs6936461 and rs7758229 on chromosome 6; rs11255841 on chromosome 10; and rs4779584, rs11632715, and rs73376930 on chromosome 15. The regions are characterized in Figs. 2, 3, 4, and 5. The pattern of associations is rather diffuse in the *STXBP5-AS1* (STXBP5 Antisense RNA 1) and *SLC22A3* genes of chromosome 6, representing the correlation among the variants in these regions (Figs. 2 and 3). Similarly, the association pattern tapers along chromosome 10. The strongest association pattern can be found on chromosome 15. This region has a more defined peak than the other regions with associations spanning two genes: *SCG5* (secretogranin V) and *GREM1* (gremlin 1, DAN family BMP antagonist).

The polygenic analysis identified 10 SNPs which appear to have a relatively strong association (i.e., large effect size) with the risk of developing colorectal cancer as can be seen in Table 2. These variants have marginal p-values between 0.19 and 1.5E−5 indicating some would have been overlooked in an standard analysis. Five of these SNPs lie in intergenic regions; three lie in introns of *ARHGEF3*, *PLCG2*, and *RGMB*; one is a deletion in *PIGN*; and one is an insertion in *SHISA9*.

**Table 2:** Polygenic risk model learned from colorectal cancer data.

Description	Chr	Position	Gene	Ref	MaF	Estimate
Intercept						0.90
Gender						0.00
Age						$-3.75$
BMI						0.00
Smoking						1.32
rs11919079	3	57086348	Intron:ARHGEF3	G	0.07	2.40
rs4888186	16	81947156	Intron:PLCG2	C	0.08	0.85
rs11016111	10	129963848	Intergenic	C	0.34	$-1.32$
rs77657157	5	98125016	Intron:RGMB	G	0.05	1.95
–	18	59822981	Deletion:PIGN	TC	0.19	$-1.39$
rs17066763	5	164113078	Intergenic	T	0.12	1.65
rs2446103	6	77328692	Intergenic	A	0.04	1.22
rs7219420	17	45800299	Intergenic	T	0.36	1.32
–	16	13018917	Insertion:SHISA9	C	0.11	1.67
rs78165118	3	12816282	Intergenic	A	0.03	2.13

Presented results include the chromosome (Chr) and position of the significant genetic variants, the gene they lie on (Gene), reference allele (Ref), minor allele frequency (MaF), and estimated effect (Estimate).

## Discussion

This preliminary study represents the first genome-wide analysis of a South Sulawesi population in Indonesia. We hope this work will motivate additional cancer research in this understudied and diverse population. Strengths of the study include the building of a colorectal cancer research program in Indonesia, the extensive questionnaire for assessing non-genetic risk factors, and genome-wide genotyping across diverse ethnicities.

Limitations of the study include the sample size due to the resource-constrained settings in Indonesia, which restricts the analysis to previously identified colorectal cancer markers and challenges shared by case-control study designs. For instance, the controls may represent different groups than cases. We attempted to account for this by frequency matching on age, sex, and ethnicity. Additionally, the timing of assessments need to be considered in interpreting the results. Given screening programs are still being developed in Indonesia, the majority of the cases had late stage colorectal cancer, stage III and IV. When BMI was assessed in these patients they already had significant weight lose, thus the direction of the effect is different than what one might expect.

Interestingly, the mean age of cases in this study was 54 which could imply a family history of cancer. Unfortunately we had limited data on family history because patients from the rural areas did not know the health history of their relatives. Indonesia also lacks a cancer registry which could also provide information on family histories of cancers. Also worth noting, the majority of the cases had rectal cancer. Recent work from Deng^14^ found that Asian countries appear to have higher rates of rectal cancer than western countries. Environmental factors are suspected to play a strong role, e.g., in this study we found that rectal cancer cases were more likely to be smokers.

For genome-wide imputation, an Indonesian population is not currently represented in common reference population such as the 1000 Genomes Project, thus some genetic markers relevant to colorectal cancer and specific to Indonesians may not impute well. However, the 1000 Genomes Projects does have samples from Vietnam. There are genomic diversity studies underway in South East Asia which may offer a suitable reference panel for Indonesians in the future^15^.

Several previously identified colorectal cancer associated SNPs replicated in this population. And we can begin characterizing these regions by examining neighboring variants. The rs7758229 variant within *SLC22A3* on chromosome 6 was originally identified and subsequently replicated in large case-control study of a Japanese population (OR of 1.3)^16^. Interestingly, in a subsequent study in a Chinese population, this SNP was not associated with colorectal cancer (OR of 0.95)^17^. However, in S. Sulawesi, we detect a statistically significant association with colorectal cancer (p = 0.009, OR of 2.2). Given these difference among East Asians, further work to understand variation in *SLC22A3* and colorectal cancer is needed. *SLC22A3* encodes for the protein OCT3, which is an organic cationic transporter. While OCT3/SLC22A3 is well characterized within neurochemistry, it has been found to play a role within oncology as well. The upregulation of SLC22A3 in head and neck squamous cell carcinoma is associated with improved prognosis while the downregulation of SLC22A3 leads to enhanced metastasis and invasion of the tumor^18^. SLC22A3 has also been implicated in the pathogenesis of prostate cancer and its expression is elevated in these neoplastic tissues^19^. The level of OCT3/SLC22A3 expression has also been linked to the level of patient responsiveness towards cancer treatments^20^; in particular, platin-based cytotoxic cancer treatments in colorectal cancer^21^ patients, as well as head and neck squamous cell carcinoma patients^18^.

Intergenic variant rs11255841 on chromosome 10 was identified in an colorectal cancer GWAS of European ancestry individuals^22^ and has replicated in a Japanese study and a large meta-analysis with nearly 37,000 cases^23,24^. With the risk allele of T, this variant had an odds ratio of 2.2 in our study, while previous reports had an odds ratio of 1.1–1.2.

The region on chromosome 15 nearby *SCG5* and *GREM1* have been flagged in multiple GWAS, e.g.,^25^. We replicated colorectal cancer associations for rs4779584 (p = 0.018), rs11632715 (p = 0.004), and rs73376930 (p = 0.010). Interestingly, the smallest p-value in the region was rs10083612 within an intron of *SCG5* (p = 1.61e−5, see Fig. 5). The role of SCG5 in colorectal cancer has not been well characterized, while much is known about its neighbor *GREM1*’s role in colorectal cancer. GREM1, which is one of the antagonists of the bone morphogenetic proteins (BMPs) found within the TGF-beta signaling pathway, has been found to be important for the survival and proliferation of several types of cancers^26^. In particular, modulated expression of *GREM1* is found in cancer-associated stromal cells. GREM1 is also found to be a proangiogenic factor, suggesting a role in cancer development when it is upregulated^27^. SCG5 and GREM1 genes have been found to be associated with polyposis syndromes that are associated with colorectal cancer^28^. A duplication that spans the 3’end of *SCG5* and the immediate, adjacent upstream region of *GREM1* is associated with hereditary mixed polyposis syndrome (HMPS) as well as tumorigenesis in juvenile polyposis. This duplication results in a 40-kb extra segment that leads to the upregulation of *GREM1* expression. The duplication is the basis for an autosomal dominant HMPS condition that is prevalent among the Ashkenazi Jewish population and is a recommended biomarker/genetic test to detect CRC in this population. Aberrant expression of *GREM1* has also been shown to underlie oncogenesis within the large intestines and colon^29^.

Two of the previously identified colorectal cancer markers replicate in this study (rs6936461 and rs9497673; see Supplementary Table 3). These SNPs are located in the intronic regions of *STXBP5-AS1* on chromosome 6. Using bioinformatics tools, it is predicted that changes from T to A in rs6936461 and A to G in rs9497673, has the potential to alter the splicing of the gene^30^. *STXBP5-AS1* is an long non-coding (lncRNA) gene. lncRNAs drive many important cancer phenotypes through their interactions with other cellular macromolecules including DNA, protein, microRNA and mRNA. The different expression of lncRNAs in colorectal cancer indicate that lncRNAs are involved in all stages of colorectal cancer. In colorectal cancer pathogenesis, lncRNAs are implicated in a variety of signaling pathways including the Wnt/-catenin signaling pathway, epidermal growth factor receptor (EGFR)/insulin-like growth factor type I receptor (IGF-IR) signaling pathway, KRAS and phosphatidylinositol-3-kinase (PI3K) pathways, transforming growth factor-beta (TGF-) signaling pathway, p53 signaling pathway, and the epithelial-mesenchymal transition (EMT) pathway^31^. While it is still unclear how *STXBP5-AS1* contributes to colon carcinogenesis, in a study involving 1067 breast cancer samples, Guo et al. identified *STXBP5-AS1* among lncRNA genes which play a role in predicting the prognostic survival with good sensitivity and specificity. The lncRNAs may act as competing endogenous RNAs (ceRNAs) and interfere in the binding of miR-190b to certain targets such as ERG, STK38L, and FNDC3A and thus contribute to breast cancer pathogenesis^32^. *STXBP5-AS1* may act similarly in colorectal cancer; it may hinder the binding of microRNAs to their target genes and subsequently modulate colorectal cancer tumorigenesis.

Interestingly, STXBP5-AS1 was identified among genes that are methylated in buccal samples in a genome-wide screen for cigarette smoke exposure, indicating its possible role in smoking-related diseases^33^. Since there is a significant difference in smoking status between cases and controls in our cohort, it is plausible that genetic variants associated with tobacco smoke are also associated with the presence of colorectal cancer in our study population.

The polygenic model represents a strategy for jointly modeling SNP effects in a GWAS and development of risk prediction models in a specific population. These models can be used to estimate an individuals risk of colorectal cancer based on easily obtainable genotypes. While most of the variants flagged in the polygenic model are novel, the gene *ARHGEF3* has been implicated in promoting nasopharyngeal carcinoma in Asians^34^. *RGMB* has been shown to promote colorectal cancer growth^35^. Additional samples will enable us to refine and validate a polygenic colorectal cancer risk model in Indonesians.

## Methods

### Study participants

Indonesia is an archipelago consisting of more than 14,000 islands. There are five major islands, and one of them is Sulawesi. Makassar is located in the southern part of Sulawesi. It is considered the largest city in eastern Indonesia. 162 colorectal cancer cases were recruited from seven hospitals throughout Makassar between 2014 and 2016. The hospitals were Wahidin Sudirohusodo Hospital, Hasanuddin University Hospital, Ibnu Sina Hospital, Akademis Hospital, Grestelina Hospital, Stella Maris Hospital, and Hikmah Hospital. 193 controls were frequency matched to cases on age category, sex, and ethnicity. Informed consents were obtained from all subjects, and all methods were carried out in accordance with the relevant guidelines and regulations as determined by ethical review approved by the Hasanuddin University Ethical Committee (registration number: UH 15040389).

### Data and DNA sample collection

Questionnaires and medical records were recorded into study data collection forms and entered into a study database. The case forms contained 382 questions and the control forms contained 319 questions. The forms included information on demographics, cancer history in the family, smoking behavior, alcohol use, and detailed dietary history. For colorectal cancer cases, the forms collected information on cancer symptoms, staging (post operation), tumor, location, histopathology, and type of surgery. The questionnaire is included as a “Supplementary file”. The database was managed by the Bioinformatics and Data Science Research Center (BDSRC) at Bina Nusantara University (Jakarta, Indonesia). A blood sample was collected from the basilic/cephalic vein on all participants for genotyping. These blood samples were stored in Hasanuddin University Laboratory at $-20^{\circ }{\text{ C }}$.

### Genotyping and imputation

DNA samples were collected at the hospital where surgery was performed (Wahidin Hospital). DNA was extracted from samples at Mochtar Riady Institute for Nanotechnology (MRIN) Laboratory https://www.overleaf.com/project/5efa1240b367400001bf3549 (Tangerang, Indonesia). Genomic DNA was extracted from 200 ${\upmu }{\text {L}}$ of whole blood sample using the QIAamp DNA Mini Kit (Qiagen, Hilden, Germany) according to the manufacturer’s protocol. DNA concentration was determined using NanoDrop ND-1000 spectrophotometer, version 3.3 (Thermo Fisher Scientific, Wilmington, DE, USA) and adjusted to a concentration of 20 ng/${\upmu }{\text {L}}$. The quality of DNA extracted was verified by purity index of OD260/OD280 (1.8–2.0) and OD260/OD230 ($>1.5$). The DNA was inspected through Gel Electrophoresis using 1% molecular biology grade Agarose (Biorad, Hercules, CA, USA). Two plates of samples (92 cases and 92 controls) were allocated for this preliminary study and filled based on the DNA quality. Extracted DNA were sent to RUCDR Infinite Biologics for genotyping (Piscataway, NJ, USA) under Material Transfer Agreement (MTA) approved by the Indonesian Health Ministry (registration number: LB.02.01/I/12749/2016).

DNA samples from study cases and controls were genome-wide genotyped on the Smokescreen Genotyping Array^36^. Using 200 ng of genomic DNA, array plates were prepared using the Axiom 2.0 Reagent Kits and then processed on the GeneTitan MC instrument (Thermo Fisher Scientific, Wilmington, DE, USA). Analysis of the raw data was performed using Affymetrix Power tools (APT) v-1.16 according to the Affymetrix best practices workflow. 183 samples remained after completing these steps. Additional steps were performed using SNPolisher to identify and select best performing probe sets and high quality SNPs for downstream analysis. 524,765 SNPs remained after QC filtering. Additional sample quality control included verifying concordance of study replicates, checking for unintentional duplicates and unexpected relatives, and verifying genetic versus reported gender. After filtering samples with missing covariates, 173 samples (84 cases and 89 controls) remained for statistical analysis.

Genome-wide imputation was performed on the Michigan Imputation Server v1.0.2^37^. Briefly, quality controlled study genotypes were reported on the forward strand and uploaded in vcf format. 1000 Genomes Phase 3^38^ was selected as a reference panel, phasing was performed using Eagle v2.3^39^, and allele frequencies were compared against the 1000 Genomes East Asian (EAS) populations. The server automatically excludes variants with alleles other than (A, C, T, G), variants with duplicate positions, indels, monomorphic sites, and allele mismatches with the reference panel.

### Statistical analysis

#### Ancestry analysis

Ancestry categories were estimated from 5515 ancestry informative markers contained on the Smokescreen Genotyping Array using fastStructure 1.0^40^. Combining study and reference data from the 1000 Genomes Project Phase 3, we estimated the ancestry proportions of East Asian (EAS), South Asian (SAS), European (EUR), and African (AFR).

#### Genome-wide association analysis

We filtered out variants with poor imputation quality ($< 0.3$) and rare variants (minor allele $< 1\%$). We then performed a marginal analysis of the remaining SNP genotype dosages fitting logistic regression models, with sex, age, body mass index, smoking status and estimated ancestries proportions (i.e., SAS,EUR,AFR) as covariates. The threshold for statistical significance in the discovery scan was set at the historical traditional genome-wide value of 5E-8. This association model was implemented using glm in R^41^.

We queried the scan results for markers previously reported to be associated with colorectal cancer. These variants were identified through previous genotyping in an independent sample of South Sulawesi colorectal cancer cases (R. Kusuma, I. Suriapranata, personal communication) and a recent catalog of colorectal cancer SNPs for a genome-wide association scan in Hispanics^42^. The source and annotation for these variants are provided in Supplementary Table 3. Variants with evidence of replication (p-value $<0.05$) were flagged for further investigation. Regional association plots were generated in LocusZoom^43^.

We also developed a polygenic model considering the joint effect of multiple genetic variants on colorectal cancer^44^. We included a screening step as a practical way to keep the number of variants under consideration in the polygenic model close to the total sample size. In this screening step the top 200 genetic associations were selected, based on Bayes factors^45^, as candidate predictors in this joint model. Bayes factors were computed for the marginal versus the null models for each SNP while controlling for gender, age, BMI, and smoking status. To jointly model these variants, we use a Bayesian variable selection technique. In particular, we fit a logistic regression model utilizing shrinkage priors for each of the explanatory variables; i.e., the covariates listed above as well as the remaining candidate SNPs. In this analysis, the generalized double Pareto shrinkage prior^46^ was specified and the parameters of the joint model were estimated via a maximum a posteriori (MAP) estimator^46^ which was obtained via an expectation-maximization (EM) algorithm^47^. The MAP estimator under these specifications simultaneously completes parameter estimation and variable selection by obtaining a sparse estimator^48^; i.e., some of the regression coefficients are estimated to be identically equal to zero thus removing the effect of the corresponding explanatory variable. The EM algorithm was developed following the techniques illustrated by Armagan et al^46^ and Polson et al^49^ and the regularization parameters were selected via the Bayesian information criterion^50^. These algorithms were implemented in R and completed within 90 s on an Intel based laptop, see Joyner et al.^44^ for details including the source code.

## Conclusions

We demonstrate replication of several colorectal cancer genetic risk factors in an Indonesian population. This study overcame the many challenges of genomic research in resource-constrained settings and provides rational for additional data collection in this population to characterize these regions more precisely and identify genetic risk factors unique to this diverse population. The primary focus of this study was replicating associations of known colorectal cancer risk variants in an Indonesian population. A secondary focus was computing genome-wide summary statistics for contributions to international colorectal cancer consortia. With additional data collections in Indonesia, we may examine and report on environmental factors (e.g., dietary factors) as well as gene–environment interactions.

## Supplementary information

Supplementary Information.
