# Supplementary material for: Genetic risk factors for colorectal cancer in multiethnic Indonesians
Source: Sci Rep. 2021 May 11;11:9988. doi: 10.1038/s41598-021-88805-4 (PMC8113452; doi:10.1038/s41598-021-88805-4)
Supplement: Supplementary file 1 — Supplementary Information. [file 41598_2021_88805_MOESM1_ESM.pdf]

Genetic Risk Factors for Colorectal Cancer in Multiethnic Indonesians

Irawan Yusuf[1, 3 *], Bens Pardamean[2, 4 * +], James W. Baurley[2 * +], Arif Budiarto[2, 5], Upik A. Miskad[1], Ronald E. Lusikooy[1], Arham Arsyad[1], Akram Irwan[1], George Mathew[3], Ivet Suriapranata[3], Rinaldy Kusuma[3], Muhamad F. Kacamarga[2, 5], Tjeng W. Cenggoro[2, 5], Christopher McMahan[6], Chase Joyner[6], and Carissa I. Pardamean[2]

[1]Faculty Medicine, Hasanuddin University, Makassar, South Sulawesi, Indonesia
[2]Bioinformatics & Data Science Research Center, Bina Nusantara University, Jakarta, DKI Jakarta, Indonesia
[3]Mochtar Riady Institute for Nanotechnology; Pelita Harapan University, Tangerang, Banten, Indonesia
[4]Computer Science Department, BINUS Graduate Program-Master of Computer Science Program, Bina Nusantara University, Jakarta, DKI Jakarta, Indonesia
[5]Computer Science Department, School of Computer Science, Bina Nusantara University, Jakarta, DKI Jakarta, Indonesia
[6]School of Mathematical and Statistical Sciences, Clemson University, Clemson, SC, USA
[*]These authors contributed equally: Irawan Yusuf, Bens Pardamean, and James W. Baurley.
[+]Corresponding Authors: bpardamean@binus.edu; baurley@binus.edu

Supplementary materials

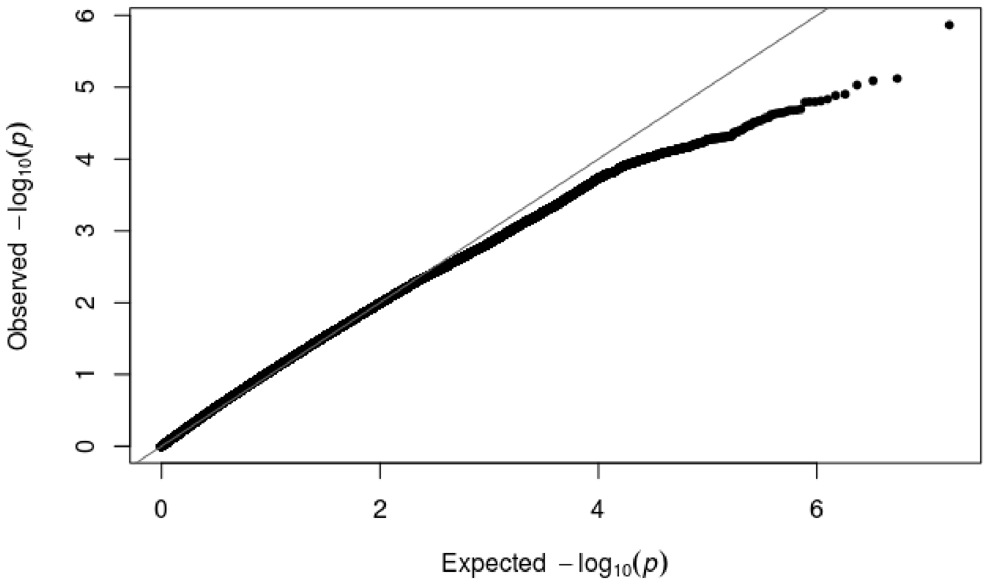

Figure 1. Observed versus expected distribution of p-values for the colorectal cancer genome-wide scan.

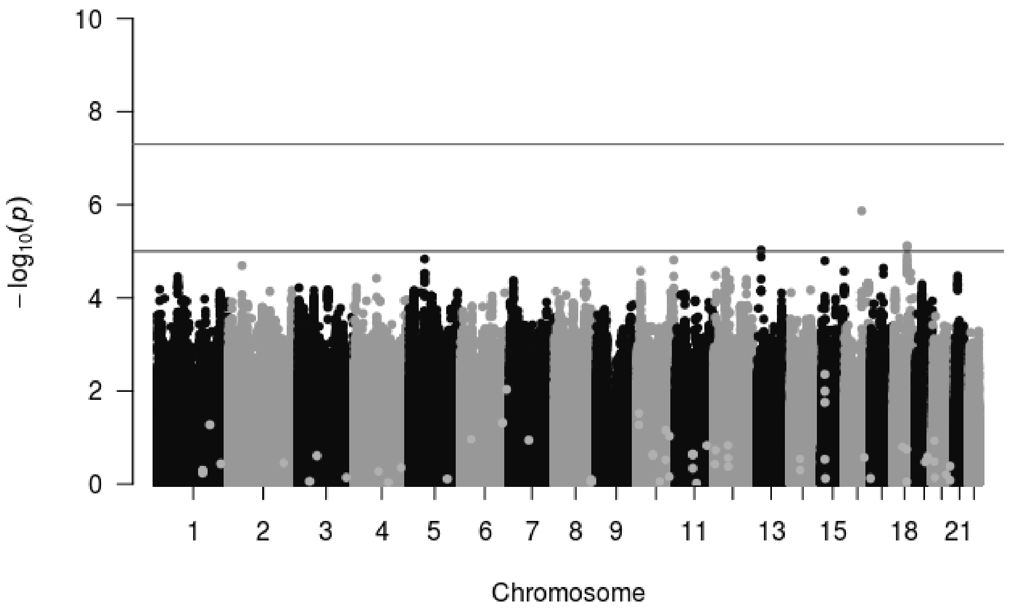

Figure 2. Manhattan plot for colorectal cancer genome-wide scan. Several SNPs were flagged with p-values < 1E-5. Green dots indicate variants flagged in previous genetic association studies.

Table 1. Results for previously identified colorectal cancer SNPs

Rsid	Gene	Chr	Pos	Ref	Alt	Source	MaF	OR	SE	P
rs12124798	Intron:RP11-451O13.1	1	157906955	A	T	(S1)	0.091	1.309	0.489	0.581
rs6684686	Intron:RP11-451O13.1	1	157909708	A	G	(S1)	0.091	1.327	0.472	0.549
rs6701170	Intron:RP11-451O13.1	1	157913954	T	C	(S1)	0.090	1.362	0.476	0.516
rs4971169	Intron:RP11-451O13.1	1	157914330	T	C	(S1)	0.088	1.372	0.486	0.516
rs10911251	Intron:LAMC1	1	183081194	A	C	(S2; S3)	0.345	0.503	0.356	0.054
rs6687758	Intergenic	1	222164948	A	G	(S4)	0.093	1.550	0.486	0.367
rs11903757	Intergenic	2	192587204	T	C	(S2)	0.161	0.713	0.364	0.353
rs35360328	Intergenic	3	40924962	T	A	(S5)	0.142	1.079	0.505	0.880
rs812481	Intron:LRIG1	3	66442435	C	G	(S5)	0.158	0.607	0.432	0.247
rs10936599	Synonymous:MYNN	3	169492101	C	T	(S4)	0.437	1.104	0.284	0.727
rs7356196	Intergenic	4	84173104	G	A	(S1)	0.309	1.229	0.329	0.532
rs3987	Intron:AC108056.1	4	118759055	A	G	(S6)	0.396	1.028	0.292	0.925
rs35509282	Intergenic	4	163333405	T	A	(S7)	0.320	0.784	0.321	0.448
rs647161	Intron:CTC-203F4.1|CTC-349C3.1	5	134499092	C	A	(S8)	0.419	0.923	0.284	0.776
rs1321311	Intron:PI16	6	36622900	C	A	(S9)	0.332	0.621	0.298	0.110
rs9497673	Intron:STXBP5-AS1	6	147281518	G	A	(S1)	0.289	1.955	0.340	0.048
rs7758229	Intron:SLC22A3	6	160840252	G	T	(S10)	0.428	2.244	0.311	0.009
rs10499807	Intergenic	7	68459734	C	T	(S1)	0.347	1.792	0.370	0.115
rs10505477	Intron:RP11-382A18.1	8	128407443	A	G	(S11; S12)	0.301	0.954	0.325	0.884
rs6983267	Intron:RP11-382A18.1	8	128413305	G	T	(S10; S11; S13; S14; S15)	0.303	0.922	0.318	0.798
rs7014346	Intron:RP11-382A18.1	8	128424792	A	G	(S4; S16)	0.254	0.966	0.350	0.922
rs10795668	Intergenic	10	8701219	G	A	(S14)	0.249	0.525	0.334	0.054
rs11255841	Intergenic	10	8739580	T	A	(S3)	0.254	0.462	0.357	0.030
rs10763129	Intron:PCDH15	10	56468045	T	G	(S1)	0.124	0.557	0.498	0.240
rs10825383	Intron:PCDH15	10	56473754	G	A	(S1)	0.124	0.560	0.488	0.234
rs704017	Intron:RP11-202P11.1	10	80819132	A	G	(S17)	0.272	0.958	0.311	0.890
rs1035209	Intergenic	10	101345366	C	T	(S3)	0.159	2.105	0.410	0.069
rs11190164	Intergenic	10	101351704	A	G	(S5)	0.251	1.460	0.367	0.303
rs12241008	Intron:VTI1A	10	114280702	T	C	(S18)	0.335	0.895	0.290	0.703
rs11196172	Intron:TCF7L2	10	114726843	G	A	(S17)	0.481	1.674	0.307	0.093
rs174537	Intron:C11orf9|RP11-467L20.9	11	61552680	G	T	(S17)	0.081	0.684	0.518	0.462
rs4246215	Utr3:FEN1	11	61564299	G	T	(S17)	0.087	0.543	0.510	0.232
rs174550	Utr5:FADS1	11	61571478	T	C	(S17)	0.087	0.543	0.510	0.232
rs1535	Intron:FADS2	11	61597972	A	G	(S17)	0.087	0.543	0.510	0.232
rs3824999	Intron:POLD3	11	74345550	T	G	(S9)	0.376	0.991	0.295	0.977
rs3802842	Intron:C11orf92|C11orf93	11	111171709	C	A	(S16)	0.324	0.651	0.298	0.150
rs10774214	Intron:RP11-264F23.3|RP11-264F23.4	12	4368352	T	C	(S8)	0.253	0.737	0.343	0.375
rs10849432	Intergenic	12	6385727	C	T	(S17)	0.130	0.562	0.437	0.187
rs34245511	Intron:LIMA1	12	50573433	G	C	(S3)	0.191	0.600	0.353	0.148
rs7136702	Intergenic	12	50880216	T	C	(S4)	0.382	1.333	0.263	0.276
rs11169552	Intergenic	12	51155663	C	T	(S4)	0.474	1.243	0.273	0.424
rs4444235	Intergenic	14	54410919	T	C	(S4; S14)	0.497	1.352	0.283	0.287
rs1957636	Intergenic	14	54560018	T	C	(S14)	0.350	1.267	0.354	0.504
rs16969681	Intergenic	15	32993111	C	T	(S14)	0.445	1.356	0.291	0.295
rs4779584	Intergenic	15	32994756	T	C	(S14; S19)	0.130	0.358	0.433	0.018
rs11632715	Intergenic	15	33004247	G	A	(S14)	0.241	4.728	0.546	0.004
rs73376930	Intron:GREM1	15	33012502	A	G	(S3)	0.446	3.010	0.428	0.010
rs1851317	Intron:RP11-814P5.1	15	35077786	A	C	(S1)	0.423	1.100	0.309	0.758
rs9929218	Intron:CDH1	16	68820946	G	A	(S4)	0.410	0.729	0.285	0.268
rs12603526	Intron:NXN	17	800593	T	C	(S17)	0.133	0.882	0.399	0.754
rs12458173	Intergenic	18	31430167	G	A	(S1)	0.309	1.845	0.436	0.160
rs7229639	Intron:SMAD7	18	46450976	A	G	(S17)	0.124	1.079	0.612	0.901
rs4939827	Intron:SMAD7	18	46453463	T	C	(S16; S20)	0.312	0.645	0.327	0.180
rs10411210	Intron:RHPN2	19	33532300	C	T	(S4)	0.153	0.705	0.365	0.337
rs1800469	Intron:TMEM91	19	41860296	A	G	(S17)	0.498	1.359	0.288	0.286
rs2241714	Nonsynonymous:B9D2	19	41869392	T	C	(S17)	0.491	1.364	0.275	0.259
rs961253	Intergenic	20	6404281	C	A	(S4; S14)	0.179	0.693	0.379	0.333
rs4813802	Intergenic	20	6699595	T	G	(S2; S14)	0.239	0.594	0.334	0.119
rs2423279	Intergenic	20	7812350	T	C	(S8)	0.416	1.106	0.287	0.725
rs6066825	Intron:PREX1	20	47340117	A	G	(S5)	0.416	0.844	0.346	0.624
rs4925386	Intron:LAMA5	20	60921044	T	C	(S2; S4)	0.260	0.758	0.338	0.414
rs2427308	Intron:CABLES2	20	60969451	C	T	(S3)	0.190	1.141	0.619	0.831

Chr: Chromosome
Pos: Chromosome Position (build 37)
Ref/Alt: Reference and alternate allele
MaF: Minor allele frequency
OR: Odds ratio
SE: Standard error

Table 2. Results from colorectal cancer genome-wide scan. Genetic variants with a marginal p-value < 1E-5.

Rsid	Gene	Chr	Pos	Ref	Alt	MaF	OR	SE	P
rs201447553	Intergenic	13	32081199	G	GT	0.218	10.746	0.536	9.36E-06
-	Intergenic	16	59740698	T	TA	0.423	17.539	0.593	1.36E-06
rs17663205	Utr3:MRO	18	48324484	G	C	0.169	0.089	0.540	7.59E-06
rs56387261	Intron:MRO	18	48325957	C	T	0.170	0.088	0.545	8.12E-06

Chr: Chromosome
Pos: Chromosome Position (build 37)
Ref/Alt: Reference and alternate allele
MaF: Minor allele frequency
OR: Odds ratio
SE: Standard error

References

[S1] Suryapranata, I. & Kusuma, R. Mochtar Riady Institute of Nanotechnology. Unpublished.

[S2] Peters, U. *et al.* Identification of genetic susceptibility loci for colorectal tumors in a genome-wide meta-analysis. *Gastroenterology* **144**, 799–807.e24, DOI: 10.1053/j.gastro.2012.12.020 (2013).

[S3] Whiffin, N. *et al.* Identification of susceptibility loci for colorectal cancer in a genome-wide meta-analysis. *Hum. Mol. Genet.* **23**, 4729–4737, DOI: 10.1093/hmg/ddu177 (2014).

[S4] Houlston, R. S. *et al.* Meta-analysis of three genome-wide association studies identifies susceptibility loci for colorectal cancer at 1q41, 3q26.2, 12q13.13 and 20q13.33. *Nat. Genet.* **42**, 973–977, DOI: 10.1038/ng.670 (2010).

[S5] Schumacher, F. R. *et al.* Genome-wide association study of colorectal cancer identifies six new susceptibility loci. *Nat. Commun.* **6**, 7138, DOI: 10.1038/ncomms8138 (2015).

[S6] Real, L. M. *et al.* A colorectal cancer susceptibility new variant at 4q26 in the Spanish population identified by genome-wide association analysis. *PLoS ONE* **9**, e101178, DOI: 10.1371/journal.pone.0101178 (2014).

[S7] Schmit, S. L. *et al.* Genome-wide association study of colorectal cancer in Hispanics. *Carcinogenesis* **37**, 547–556, DOI: 10.1093/carcin/bgw046 (2016).

[S8] Jia, W.-H. *et al.* Genome-wide association analyses in east asians identify new susceptibility loci for colorectal cancer. *Nat. genetics* **45**, 191 (2013).

[S9] Dunlop, M. G. *et al.* Common variation near CDKN1A, POLD3 and SHROOM2 influences colorectal cancer risk. *Nat. Genet.* **44**, 770–776, DOI: 10.1038/ng.2293 (2012).

[S10] Cui, R. *et al.* Common variant in 6q26-q27 is associated with distal colon cancer in an Asian population. *Gut* **60**, 799–805, DOI: 10.1136/gut.2010.215947 (2011).

[S11] Zanke, B. W. *et al.* Genome-wide association scan identifies a colorectal cancer susceptibility locus on chromosome 8q24. *Nat. Genet.* **39**, 989–994, DOI: 10.1038/ng2089 (2007).

[S12] Gruber, S. B. *et al.* Genetic variation in 8q24 associated with risk of colorectal cancer. *Cancer biology & therapy* **6**, 1143–7 (2007).

[S13] Haiman, C. A. *et al.* A common genetic risk factor for colorectal and prostate cancer. *Nat. Genet.* **39**, 954–956, DOI: 10.1038/ng2098 (2007). NIHMS150003.

[S14] Tomlinson, I. P. *et al.* A genome-wide association study identifies colorectal cancer susceptibility loci on chromosomes 10p14 and 8q23.3. *Nat. Genet.* **40**, 623–630, DOI: 10.1038/ng.111 (2008).

[S15] Hutter, C. M. *et al.* Characterization of the association between 8q24 and colon cancer: Gene-environment exploration and meta-analysis. *BMC Cancer* **10**, 670, DOI: 10.1186/1471-2407-10-670 (2010).

[S16] Tenesa, A. *et al.* Genome-wide association scan identifies a colorectal cancer susceptibility locus on 11q23 and replicates risk loci at 8q24 and 18q21. *Nat. Genet.* **40**, 631–637, DOI: 10.1038/ng.133 (2008). NIHMS150003.

[S17] Zhang, B. *et al.* Large-scale genetic study in east asians identifies six new loci associated with colorectal cancer risk. *Nat. genetics* **46**, 533 (2014).

[S18] Wang, H. *et al.* Fine-mapping of genome-wide association study-identified risk loci for colorectal cancer in African Americans. *Hum. Mol. Genet.* **22**, 5048–5055, DOI: 10.1093/hmg/ddt337 (2013).

[S19] Jaeger, E. *et al.* Common genetic variants at the CRAC1 (HMPS) locus on chromosome 15q13.3 influence colorectal cancer risk. *Nat. Genet.* **40**, 26–28, DOI: 10.1038/ng.2007.41 (2008).

[S20] Broderick, P. *et al.* A genome-wide association study shows that common alleles of SMAD7 influence colorectal cancer risk. *Nat. Genet.* **39**, 1315–1317, DOI: 10.1038/ng.2007.18 (2007).
